# Characterization of a New Immunosuppressive and Antimicrobial Peptide, DRS-DA2, Isolated from the Mexican Frog, *Pachymedusa dacnicolor*

**DOI:** 10.1155/2024/2205864

**Published:** 2024-01-13

**Authors:** Claire Lacombe, Estefania Aleman-Navaro, Thierry Drujon, Veronica Martinez-Osorio, Emmanuelle Sachon, Erika Melchy-Pérez, Ludovic Carlier, Lorena Elizabeth Fajardo Brigido, Yannick Fleury, Christophe Piesse, Guadalupe Gutiérrez-Escobedo, Alejandro De las Peñas, Irene Castaño, Florie Desriac, Jose Luis Beristain-Hernandez, Christophe Combadiere, Yvonne Rosenstein, Constance Auvynet

**Affiliations:** ^1^Laboratoire des Biomolécules, LBM, Sorbonne Université, École Normale Supérieure, PSL University, CNRS, Paris, France; ^2^Faculté des Sciences, Université Paris Est-Créteil Val de Marne, Créteil, France; ^3^Instituto de Biotecnología, Universidad Nacional Autónoma de México, Cuernavaca, Morelos, Mexico; ^4^Posgrado de Ciencias Bioquímicas, Universidad Nacional Autónoma de México, Mexico City, Mexico; ^5^Plateforme MS3U Mass Spectrometry Sciences Sorbonne University, Fédération de Chimie Moléculaire de Paris Centre, FR2769, Sorbonne Université, Paris, France; ^6^LUBEM EA 3882, IUT Quimper, Université de Bretagne Occidentale, Quimper, France; ^7^Plateforme de Synthèse Peptidique, Institut de Biologie Paris-Seine (ISBS), Sorbonne Université, CNRS, Paris, France; ^8^IPICYT, División de Biología Molecular, Instituto Potosino de Investigación Científica y Tecnológica, San Luis Potosí, Mexico; ^9^Hepatobiliary and Pancreatic Surgery Clinic, General Surgery Department La Raza National Medical Center, Instituto Mexicano del Seguro Social, Ciudad de México, Mexico; ^10^Sorbonne Université, Institut National de Santé et de Recherche Medicale (Inserm), Centre National de la Recherche Scientifique (CNRS), Centre d'Immunologie et des Maladies Infectieuses, Cimi-Paris, Paris, France

## Abstract

Inflammatory and antimicrobial diseases constitute a major burden for society, and fighting them is a WHO strategic priority. Most of the treatments available to fight inflammatory diseases are anti-inflammatory drugs, such as corticosteroids or immunomodulators that lack cellular specificity and lead to numerous side effects. In addition to suppressing undesired inflammation and reducing disease progression, these drugs lessen the immune system protective functions. Furthermore, treating infectious diseases is more and more challenging due to the rise of microbial resistance to antimicrobial drugs. Thus, controlling the inflammatory process locally without compromising the ability to combat infections is an essential feature in the treatment of inflammatory diseases. We isolated three forms (DRS-DA2N, DRS-DA2NE, and DRS-DA2NEQ) of the same peptide, DRS-DA2, which belongs to the dermaseptin family, from the Mexican tree frog *Pachymedusa dacnicolor*. Interestingly, DRS-DA2N and DRS-DA2NEQ exhibit a dual activity by inducing the death of leukocytes as well as that of Gram-negative and Gram-positive bacteria, including multiresistant strains, without affecting other cells such as epithelial cells or erythrocytes. We showed that the death of both immune cells and bacteria is induced rapidly by DRS-DA2 and that the membrane is permeabilized, leading to the loss of membrane integrity. We also validated the capacity of DRS-DA2 to regulate the pool of inflammatory cells *in vivo* in a mouse model of noninfectious peritonitis. After the induction of peritonitis, a local injection of DRS-DA2N could decrease the number of inflammatory cells locally in the peritoneal cavity without inducing a systemic effect, as no changes in the number of inflammatory cells could be detected in blood or in the bone marrow. Collectively, these data suggest that this peptide could be a promising tool in the treatment of inflammatory diseases, such as inflammatory skin diseases, as it could reduce the number of inflammatory cells locally without suppressing the ability to combat infections.

## 1. Introduction

Considering that chronic inflammation plays a central role in the development of the majority of chronic-degenerative diseases, but also of some infectious diseases, including acute respiratory distress syndrome, a common complication of various viral pneumonia caused, for example, by coronaviruses, such as MERS-CoV, SARS-CoV, and recent SARS-CoV-2 [1], it is of great interest to identify new drug candidates to specifically control the inflammatory process without compromising the ability of the body to combat infections. Indeed, most of the anti-inflammatory drugs available treat inflammatory diseases systemically and thus compromise the ability of the immune system to combat infections [2, 3]. Antimicrobial peptides (AMPs), produced by skin and mucosal cells, among others, are fully part of the innate immune system [4, 5]. Originally identified for their microbicidal properties by killing a large scale of Gram-negative and Gram-positive bacteria, fungi, and protozoa mainly by permeabilizing their membrane, AMPs are now recognized to modulate the immune system [6]. Among all the organisms producing peptides, AMPs isolated from frogs present a broad range of interesting biological activities in mammals. Notably, a number of these AMPs have been identified to modulate inflammatory processes by promoting the migration of immune cells through the activation of chemotactic receptors and/or by stimulating the production of proinflammatory or anti-inflammatory cytokines [6], while exhibiting antimicrobial properties against a wide range of bacteria.

The aim of this study was to isolate and characterize, *in vitro* and *in vivo*, a peptide that could be an alternative candidate in the treatment of inflammatory diseases by regulating the pool of inflammatory cells locally, rapidly, and for a determined period of time. Thus, we tested the potential of skin exudate extracted from the Mexican tree frog *Pachymedusa dacnicolor* to present an immunosuppressor effect. We found a fraction that regulated the pool of inflammatory cells by specifically inducing the death of immune cells. Within that fraction, we isolated three forms of the same peptide, differing only by the deletion of one or two residues from the C-terminus. The longer peptide (DRS-DA2NEQ) corresponds to the putative sequence determined from cDNA, published in 1998 by Wechselberger [7], but the corresponding peptide was not isolated. As these sequences were rather close to a family of antimicrobial peptides, dermaseptins, we also proceeded to test their antimicrobial activity. We synthesized the two extreme forms of this peptide (DRS-DA2NEQ and DRS-DA2N) and were able to demonstrate that, *in vitro*, these peptides killed, at micromolar concentrations, Gram-negative and Gram-positive bacteria, including multiresistant strains, different *Candida* strains, and human and mouse leukocytes, without affecting other cells such as primary and transformed epithelial cells or erythrocytes. In all cases, the shorter peptide (DRS-DA2N) seemed to be more potent in inducing the death of immune cells and bacteria than the longer one (DRS-DA2NEQ). We showed that DRS-DA2 induced the death of both immune cells and bacteria by rapidly inducing the permeabilization of the membrane. Finally, we validated the capacity of this peptide to induce specifically the death of inflammatory cells *in vivo* in a murine model of inflammatory disease, noninfectious peritonitis, and showed that the short form DRS-DA2N exhibited a local, and not systemic, immunoregulatory effect by lessening the number of leukocytes in the peritoneal cavity after induction of peritonitis.

## 2. Materials and Methods

### 2.1. Animals

All experiments involving living beings were carried out according to the guidelines of the Bioethics Committee of the Instituto de Biotecnología, UNAM (bioethics approval project 334/336 Pequeños péptidos de defensa: nuevas moléculas con capacidades inmunomoduladoras y antimicrobianas), and by authorized investigators. Specimens of *Pachymedusa dacnicolor* were captured in the state of Morelos (Mexico) and housed as previously described [8]. 10-week-old male or female C57BL/6 or Balb/c mice were kept in pathogen-free conditions with food and water available *ad libitum* and housed in a 12 h light/12 h dark (100–500 lux) cycle.

### 2.2. Peptide Purification and Identification by Mass Spectrometry

200 *µ*L of fresh skin exudate was recovered by gently squeezing the lateral-dorsal portion of the frog's skin, as described [8]. For identification by mass spectrometry, samples were prepared according to the “dried-droplet” method [9] with CHCA (*α*-cyano-4-hydroxycinnamic acid, Applied Biosystems) (5 mg/mL) as the matrix in ACN/aqueous 0.1% TFA (1 : 1). MALDI-TOF experiments were performed using a 4700 proteomic analyzer apparatus (Applied Biosystems, Courtaboeuf, France) fitted with a pumped diode laser Nd:YAG: *λ* = 355 nm, a pulse duration of 4 ns, and a repetition rate of 200 Hz. MALDI-TOF/TOF experiments were performed under CAD (N_2_, 5.3 × 10^−5^ Pa) at 1 keV (collision energy). Precursor ion window is +/- 2u. MS and MS/MS experiments were externally calibrated using classical peptides mixtures.

### 2.3. Solid-Phase Peptide Synthesis

Peptides were synthesized by automated solid-phase peptide synthesis using Fmoc/tBu chemistry on Activo-P11 (Activotec) and were then purified by HPLC (Waters, C18 column). Identities of the peptides were validated by MALDI-TOF mass spectrometry (MS Voyager Applied Biosystems). Considering the presence of one tryptophan residue in the sequence, concentrations were determined by UV spectroscopy (Nanodrop, Labtech.com) assuming an extinction coefficient for tryptophan *ε*_280_ = 5 600 M^−1^·cm^−1^.

### 2.4. Circular Dichroism (CD) Spectroscopy

The CD spectra of the peptides were recorded as previously described [10]. Measurements were carried out in 10 mM Tris buffer at pH 7.4 and in the presence of POPC, POPG, and POPG-POPC (25 : 75) LUVs or POPG-POPE (25 : 75) LUVS to mimic *E. coli* inner membrane composition at a peptide/lipid molar ratio of 1/50. Dichroic spectra were corrected by subtracting the background obtained for each peptide-free solution. An analysis of CD spectra was conducted after smoothing, and conversion to molar ellipticity was calculated as follows: *θ* = *θobs*/(*10xnxl*), where *θobs* is the observed ellipticity, *n* is the number of bonds, *c* is the peptide concentration in mol. L^−1^, and *l* is the path length in cm.

### 2.5. NMR Spectroscopy and Structure Calculations

The structure of DRS-DA2N bound to SDS micelles was determined by solution-state NMR spectroscopy as previously described [11]. In brief, 2D ^1^H-^1^H TOCSY, 2D ^1^H-^1^H NOESY (80 ms mixing time), and 2D ^1^H-^13^C HSQC experiments were recorded at 40°C on a Bruker 500 MHz Avance III spectrometer equipped with a 5 mm TCI cryoprobe. NMR samples contained 800 *µ*M DRS-DA2N and 80 mM SDS-d25 (Eurisotop) in either 600 *µ*L H_2_O/D_2_O (95 : 5) or 100% D_2_O. 324 interproton distance restraints (121 intraresidual, 102 sequential, and 95 medium-range) were derived from NOESY cross-peak intensities, and 50 dihedral angle restraints (25 Φ and 25 Ψ) were predicted by TALOS-N from ^13^C_*α*_, ^13^C_*β*_, ^1^H_*α*_, and ^1^H_N_ chemical shifts (Table S1) [11]. Structures were calculated by torsion angle dynamics in CNS and refined in an explicit layer of water including an electrostatic energy term in the energy function [12].

### 2.6. Cell Preparation

Human leukocytes were provided by the « Banco de Sangre del Hospital Regional del IMSS » and the Centro Estatal de la Transfusión Sanguínea in Cuernavaca, Morelos, Mexico. Institutional committees approved the acquisition and isolation of human peripheral blood leukocytes. Human leukocytes and erythrocytes were obtained as previously described [8]. Mouse bone marrow cells (MBMCs) and erythrocytes were obtained from C57BL/6 or Balb/c mice [13]. Mouse epithelial cells were isolated from C57Bl/6 mice, and human epithelial cells were recovered from surgery residues. In brief, intestinal tissues were minced and digested in 450-U/mL collagenase I, 125-U/mL collagenase XI, 60-U/mL hyaluronidase, and 60-U/mL DNAse for 1 hour before gradient density centrifugation on Histopaque-1083 (Sigma-Aldrich, St. Louis, MO). A549 cells (ATCC CCL-185), a human lung carcinoma cell line, were cultivated at 37°C in a humid atmosphere and 5% CO_2_, with DMEM advanced medium (Gibco®) supplemented with 5% of fetal bovine serum (FBS), 2 mM of glutamine, 100 U of penicillin, and 50 *μ*g/mL of streptomycin (Gibco®). Jurkat E6-1 cells were cultured in an RPMI medium supplemented with 5% fetal bovine serum.

### 2.7. Cytotoxic Assay

5.10^5^ mouse or human leukocytes or primary epithelial cells, A549 or Jurkat E6-1 cells, were incubated in 100 *µ*l RPMI or DMEM containing 0.5% BSA and 10 nM HEPES with the peptides at different concentrations for 2 hours at 37°C, 5% CO_2_. Incubation of the cells with DMSO 30% was used as a positive control. For the kinetic assay, the cells were incubated with the peptides for different time periods. Viability of leukocytes and Jurkat cells was determined by adding a viability dye (eFluor 780) and counted by FacsCanto flow cytometry with a predetermined number of beads (Polybead, Carboxylate Microspheres, Polysciences, Inc., Le Perray-en-Yvelines, France) added to the samples. Mouse leukocytes were stained with anti-mouse Ly6G-PE, anti-mouse NK1.1-PE, anti-mouse CD11b-PerCP, and anti-mouse F4/80-APC (BD Biosciences, Rungis, France). Human leukocytes were stained with anti-human CD45-PB, anti-human CD16-PE-Cy7, anti-human CD56-PE-Cy7, anti-human CD14-FITC, anti-human CD3V500, anti-human CD8-APC, and anti-human CD4-PE. Data were analyzed with FlowJo.

For the cell line A549, viability was assessed by the CCK8 assay (DoJindo) as indicated by the manufacturer. In brief, 100 *µ*L of cell suspension was dispensed (12 500 cells/well) in a 96-well plate. After 24-hour preincubation in a humidified incubator (37°C, 5% CO_2_), 10 *µ*L of various concentrations of peptides was added, and the plaque was incubated for 1 h in the incubator. 10 *µ*L of CCK-8 solution was then added to each well of the plate. The absorbance at 450 nm was measured after 3 h incubation.

### 2.8. Hemolytic Tests

Blood from a mouse or healthy adult donor was centrifuged at 800 × *g* for 10 minutes at 4°C. The pellet containing red blood cells was then washed three times in 10 volumes of PBS and was diluted in 4% (v/v) in PBS. Blood and peptides at different concentrations were then incubated at 37°C for one hour. Cells were centrifuged 1500 × *g* for 5 minutes at room temperature. Hemolysis was measured in the supernatant at 550 nm. Red blood cells lysed with 1% Triton X-100 was used as a control, achieving 100% hemolysis. Percent hemolysis was calculated using the following formula: % hemolysis = 100% × [(*A* − *A*0)/(*At* − *A*0)], where *A* represents the absorbance of the peptide sample at 550 nm and *A*0 and At represent 0% and 100% hemolysis determined in 10 mM PBS and 0.1% Triton X-100, respectively.

### 2.9. Antimicrobial Assays

The minimum inhibitory concentration (MIC) was determined by a liquid test in 96-well microplates as previously described [13]. 100 *µ*L of the sample to be tested was distributed in triplicate to obtain a range of final concentrations from 100 to 0.2 *µ*M in the wells. 90 *µ*L of bacterial suspension (5 × 10^5^ CFU/mL for Gram-negative bacteria and 5 × 10^5^ CFU/mL for Gram-positive strains in Mueller–Hinton broth) were added to each well [14]. The microtiter plates were incubated at 37°C overnight. The optical density of the wells was measured at 620 nm, and the results were interpreted by calculating the percentage of growth in each well according to the formula: % growth = (OD_600_ well − OD_600_ T0)/(OD_600_ of T100 − OD_600_ T0) *∗* 100, where OD_600_ T100 = DO_600_ growth control (bacterial suspension without the test sample, 100% growth), OD_600_ T0 = OD_600_ of sterility control (culture medium + bacteria + 10 *µ*L formaldehyde, 0% growth) well, and OD_600_ = OD_600_ of the well which was desired to calculate the percentage of growth (+ bacterial suspension test sample). MIC is the minimum concentration of the peptide that inhibits bacterial growth after incubation at 37°C for 20 hours.

The minimum bactericidal concentration (MBC) is defined as the minimum concentration of the peptide killing 99.9% of bacteria after incubation at 37°C for 20 hours. This test consisted of the spreading of the contents of the wells where the peptide inhibited bacterial growth on a Petri dish (MH agar), followed by incubation for 24 h at 37°C.

### 2.10. Antifungal Assay: Strains and Growth Conditions

The fungal pathogens *C. glabrata* standard strain CBS138 [15] and *C. albicans* strain SC5314 [16] were grown in rich (YPD) media. Yeast extract-peptone-dextrose (YPD) medium was prepared, contained yeast extract at 10 g/L and peptone at 20 g/L, and was supplemented with 2% glucose. *C. albicans* and *C. glabrata* cells were grown for 48 h at 30°C and diluted into fresh YPD media containing different concentrations of each peptide (DRS-DA2N and DRS-DA2NEQ): 1.5, 3, or 15 *µ*M. These cultures were grown in a Bioscreen C apparatus with constant shaking for 1,500 min, and OD_600_ was determined every 15 min as described previously [17].

### 2.11. Time Kill

Time-killing experiments were achieved according to [18, 19] with minor modifications. The peptide's concentration was 1 × MIC and 2 × MIC. At the time points of 15, 30, 45, and 60 min and after homogenization, suspensions were diluted in 10 mM sodium phosphate buffer (pH 6.8) and spread onto MH agar plates. After overnight incubation at room temperature, CFUs were counted.

### 2.12. *In Vitro* Peptide Stability

One mL of RPMI supplemented with 25% (v/v) rat serum was equilibrated at 37°C for 15 min before adding the peptide (final concentration of 300 *µ*M). At different time points, 300 *µ*L of the reaction solution was removed and completed to 600 *µ*L with 96% ethanol. The sample was cooled at 4°C for 15 min and then spun to precipitate serum proteins. The supernatant was recovered and submitted to the viability assay as described in the cytotoxic assay section or analyzed by RP-HPLC on C18–300 100 × 4, 6 mm. A linear gradient from 100% buffer A (0.1% TFA in H_2_0) to 60% of buffer B (0.1% TFA in ACN) was used over 10 min with a flow rate of 1 mL/min. The absorbance was detected at both 220 nm and 280 nm.

### 2.13. Thioglycolate-Induced Peritoneal Inflammation

C57BL/6 mice were injected intraperitoneally with 1 mL 3% (wt/vol) thioglycolate (Sigma-Aldrich, l'Ile d'Abeau, France) dissolved in sterile NaCl and then 14, 19, and 24 hours later injected i.p. with DRS-DA2N or DRS-DA2NEQ (15 *µ*g/injection). NaCl was used as a negative control. Twelve hours after the last injection, mice were killed, and 3 mL of cold PBS was injected i.p. to harvest peritoneal cells, which were then stained with anti-mouse Ly6G-PE, anti-mouse NK1.1-PE, anti-mouse CD11b-PerCP, and anti-mouse F4/80-APC (BD Biosciences, Rungis, France). Blood and bone marrow were also collected and stained with the same antibodies. Cells and beads were then counted by FACSCalibur flow cytometry, and data were analyzed with FlowJo. Cells being CD11b^hi^Ly6G^−^NK1.1^−^ are considered to be monocytes (Mo), CD11b^hi^Ly6G^−^NK1.1^−^ F4/80^+^ macrophages (MΦ), and CD11b^hi^Ly6G^+^NK1.1^−^ neutrophils (PMN).

### 2.14. Apoptosis/Necrosis Assays

Induction of apoptosis or necrosis by the peptides was tested by incubating 5.10^5^ Jurkat E6-1 cells in 200 *µ*l RPMI containing 10% FBS and 10 nM HEPES with the peptides at 5 *µ*M for 2 hours at 37°C, 5% CO_2_. A positive control was obtained by exposing the cells to UV light for 10 min (1 mJ/cm^2^, Stratagene UV Stratalinker 2400), before incubating them for 4 hours at 37°C, 5% CO_2_, RPMI 10% FBS. Cells were then washed, centrifuged, resuspended, and incubated 30 min at 4°C in the dark with Viability dye 780 (BioLegend). After washing the cells, Annexin-V-FITC in annexin-binding buffer was added, and the cells were incubated for 15 minutes at room temperature in the dark. The cells were fixed with PFA at a final dilution of 2% before being analyzed by FACSCanto flow cytometry.

### 2.15. Peptide-Induced Permeabilization of the Inner Bacterial Membrane [20]

Permeabilization of the *E. coli* ML35p membrane by both peptides was assayed by measuring the *β*-galactosidase activity with the chromogenic substrate o-nitrophenyl-*β*-D-galactopyranoside (ONPG). Bacteria were grown in 10% Mueller–Hinton broth with 50 *µ*g/mL ampicillin, washed twice with the test buffer (10 mM phosphate, 100 mM NaCl at pH 7.4 and 300 *µ*g/mL Mueller–Hinton broth), and diluted in the test buffer to an absorbance of 0.6 at 630 nm (mid-log phase). 50 *µ*L aliquots of bacterial suspension were mixed with 25 *µ*L pf the peptide at various concentrations and 25 *µ*L ONPG (2 mM, i.e., 0.5 mM final) in a 96-well plate. The hydrolysis of ONPG was monitored by measuring the absorbance of released o-nitrophenol at 412 nm with a Fluostar Galaxy microplate reader (BMG LabTech, France) [8]. Data shown are the means of 3 independent experiments realized in duplicate.

### 2.16. Membrane Depolarization

*E. coli* cells were grown at 37°C to the mid-logarithmic phase and were collected by centrifugation (4000 x g, 10 min), before being resuspended in MH with 0.1 mg/mL BSA and were diluted in the same buffer to an OD_620_ of 0.6. The bacterial suspension was divided into 125 *µ*L aliquots in 96-well microplates. 10 *µ*L of DiSC_3_(5) in 1% DMSO was added in each well to a 10 *µ*M final concentration. After 10 minutes, 10 *µ*L of EDTA (15 mM final concentration) was added. After recording the baseline fluorescence, 5 *µ*L of the aqueous peptide stock was added to the desired final concentration. The increase in the fluorescence intensity registers dye release upon reduction of the transmembrane potential. The fluorescence reading was monitored by using a microplate reader (BMG Fluostar) at an excitation wavelength of 620 nm and an emission wavelength of 670 nm.

### 2.17. Preparation of Vesicles

The lipids 1-palmitoyl-2-oleoyl-sn-glycero-3-phospho-(1′-rac-glycerol) (POPG) and 1-palmitoyl-2-oleoyl-sn-glycero-3-phosphoethanolamine (POPE) were obtained from Avanti Polar Lipids, Inc., and used without further purification. Desired mixtures of phospholipids were first dried in glass tubes under nitrogen and then maintained under reduced pressure for at least 60 min. Lipid films were subsequently hydrated in PBS to yield a lipid concentration. The resulting dispersions were subjected to 5 freeze-thaw cycles by alternately placing the sample vial in a liquid nitrogen bath and a warm water bath (40°C). The large multilamellar vesicles (MLVs) were then extruded 11 times through polycarbonate membranes (100 nm pore size; Whatman, Maidstone, UK) on an Avanti mini-extruder apparatus (Avanti Polar Lipids, Inc., Alabaster, AL) equipped with polycarbonate membranes (at 100 and 400 nm cutoff) to generate large unilamellar vesicles (LUVs).

### 2.18. ANTS/DPX Leakage Assay

The vesicles of POPE/POPG (75/25) at 25 mM concentration were obtained as previously described and suspended in a solution of 12.5 mM ANTS and 45 mM DPX in 10 mM HEPES. After freeze-thaw and extrusion, LUVs were separated from external ANTS/DPX by ultracentrifugation (3 × 50 000 rpm, 15 min). Final lipid concentration was measured by the method of Rouser.

To measure the leakage of DPX, the vesicles were diluted to a concentration of 0.5 mM lipid. The peptide was added to individual samples at concentrations from 0.5 *μ*M (*P*: *L* = 1 : 1000) to 50 *μ*M (*P*: *L* = 1 : 10). ANTS fluorescence was monitored using a fluorimeter (excitation = 350 nm; emission = 530 nm) or a fluorescence plate reader (excitation filter wavelength/bandpass = 340/11; emission 530/25.

When leakage has stopped or slowed significantly (generally 10–60 minutes), the final ANTS fluorescence was measured. Fractional leakage was calculated as (*F*_sample_ − *F*_initial_)/(*F*_lysed_−*F*_initial_).

### 2.19. Intrinsic Tryptophan Fluorescence

The fluorescence spectra of DRS-DA2 peptides were recorded from 295 to 475 nm with excitation at 280 nm in a spectrofluorometer using a quartz cuvette (Jobin Yvon Horiba spectrometer H 10–61 UV, Kyoto, JAP; Biologic Science Instruments ALX 250, MM 450, PMS 250; Software: Bio-Kine 32 V4.60, Grenoble, F). 150 *μ*L of a 20 *μ*M peptide solution was placed in a 10 × 2 mm cuvette (Hellman Analytics, Müllheim, GER), and increasing amounts of the lipid solution were added to obtain lipid concentrations of 10, 25, 50, 100, 200, 500, 1500, and 3000 *μ*M. After each addition, the tryptophan residue of the peptide was excited at 280 nm, and the emission spectra were recorded from 300 to 480 nm. The intensity of the emission was first plotted against the wavelength of the emitted fluorescence of tryptophan. Taking into consideration that the peptide's concentration changes during the experiment because of the addition of amounts of lipid-containing buffer, the data were formatted so that all information corresponds to the same peptide concentration. The subsequent normalization of the graphs allows for an easy comparison of the results from different experiments. We chose to calculate Δ*λ*_max_—hence the difference between *λ*_max_ at any concentration of lipid and the *λ*_max_ of the peptide solution without added liposomes—and to plot the values against the ratio (concentration of lipid)/(concentration of peptide). An apparent binding constant equivalent to the concentration of lipid at which the lipid-induced blue shift was half-maximal (*K*_*L*_) was thus determined.

## 3. Results

Isolation of a new peptide belonging to the dermaseptin family with immunosuppressive properties.

**Figure 1:**
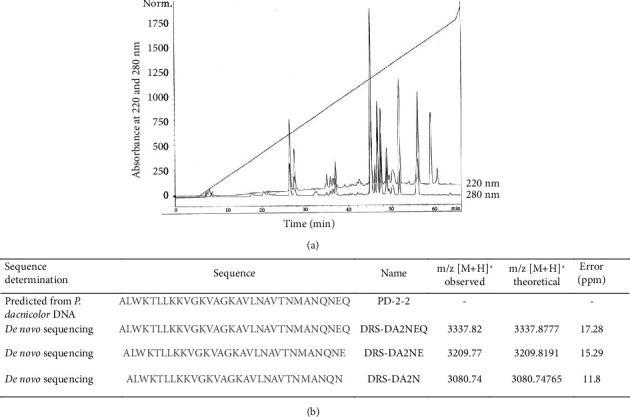
Isolation and purification of a new immunosuppressor peptide, dermaseptin DRS-DA2. (a) Fraction I that was shown to induce the death of leukocytes was purified by RP-HPLC. Among all the fractions tested for an immunosuppressive effect, the one with a retention time of 49 min (arrow) induced the death of leukocytes. (b) *De novo* characterization by MALDI-TOF/TOF mass spectrometry identified three peptides in fraction 49: DRS-DA2NEQ, DRS-DA2NE, and DRS-DA2N. Those peptides were all carboxylated at their C-terminus. Isotopic masses are given for the protonated molecules, and mass errors are given in ppm. The sequence of the nontruncated peptide corresponds to PD-2-2.

As previously reported [11], *Pachymedusa dacnicolor* skin exudate was extracted and purified to homogeneity by a two-step protocol. In brief, 1.1 mL of skin exudate recovered by gently squeezing the skin parotid glands was first fractionated on a Sephadex G-50 column (Fig S1), and the three fractions obtained were incubated with human leukocytes at 37°C, 5% CO_2_. After 30 min of incubation with fraction I at a concentration of 100 *µ*g mL^−1^, 100% of the cells were dead (insert Fig S1). High molecular weight fraction I was then purified by RP-HPLC (Figure 1(a)). Among all fractions incubated with leukocytes, the fraction with a retention time of 49 min was found to induce the death of leukocytes (data not shown). This RP-HPLC fraction was further analyzed by mass spectrometry for peptide identification and *de novo* sequencing. The MALDI-TOF/TOF analysis of the RP-HPLC fraction revealed three peptides corresponding to monoprotonated ions [M + H]^+^ with at m/z 3337.82, 3209.77, and 3080.74 for the first isotope (monoisotopic masses), respectively, and consistent with dermaseptin-like peptides (Figure 1(b)). The three peptides, identified with mass precision in the 10–20 ppm range, only differ from each other by the loss of one residue (*Q*) or two residues (EQ) at their C-terminus. The nontruncated sequence, which corresponds to the sequence of DRS-DA2NEQ, had been predicted from frog DNA in 1998 [7] but never confirmed until now, as the corresponding peptide was not isolated. These peptides were named DRS-DA2N, DRS-DA2NE, and DRS-DA2NEQ (Figure 1(b)). Synthetic peptides were used for their characterization.

**Figure 2:**
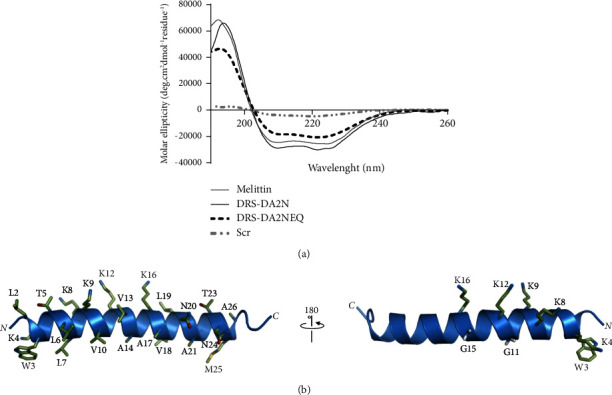
Structural properties of DRS-DA2N and DRS-DA2NEQ. (a) Far-UV CD spectra of DRS-DA2N, DRS-DA2NEQ, melittin, and Scr in POPE: POPG (75 : 25) LUVs. Data shown are representative of at least three different experiments. (b) NMR structure of DRS-DA2N bound to SDS micelles (ribbon representation of the lowest-energy conformer). Side chains of residues forming the polar and hydrophobic faces are represented by sticks.

DRS-DA2 peptides were then structurally characterized by circular dichroism (CD) and NMR. Antimicrobial peptides are generally unstructured in aqueous solution and tend to adopt a well-defined conformation in contact with membranes. CD spectra were recorded in the presence of Tris buffer (10 mM) or negatively charged LUVs composed of POPE and POPG phospholipids, with a 3 : 1 ratio that reflects the PE/PG ratio in the inner membrane of *E. coli*. In the presence of negatively charged LUVs, both DRS-DA2N and DRS-DA2NEQ adopted dichroic spectrum characteristics of an *α*-helix conformation, like the one obtained for melittin, a well-known pore-forming peptide [21, 22], as indicated by the two intense negative bands detected around 208 and 222 nm. However, a difference in the percentage of helicity was observed between the peptides, indicating that DRS-DA2N (91%) seemed to be better structured than DRS-DA2NEQ (62%) and melittin (77%). As expected, Scr (KGNVLVKAAMLTKVLAKNKGVQNANALTW), the random form of DRS-DA2N, remained unstructured (Figure 2(a)). On the contrary, in the presence of Tris buffer, all peptides remain unstructured (Fig.  S2). The conformation of DRS-DA2N was further characterized at the residue level by solution-state NMR in the presence of SDS micelles that mimic the amphipathic properties of the lipid bilayer. Analysis of NMR data indicated that the region from L2 to A26 adopted a well-defined helical structure, while the terminal residues A1 and N27-N29 are rather unstructured (Fig.  S3). The structure of DRS-DA2N exhibited a pronounced amphipathic character with the hydrophobic face predominantly formed by A, L, and V residues and the hydrophilic face containing 4 K residues (K8, K9, K12, and K16) (Figure 2(b)). The interfacial position of residue K4 allowed the ammonium group of its long-side chain to potentially interact with the phospholipid head groups at the surface of the bacterial membrane or to make *π*-cation interactions with the aromatic group of W3 on the hydrophobic face of the helix. Despite its weak helical propensity, the presence of two G residues (G11 and G15) in a GXXXG motif did not interrupt the helical conformation in the central region of the peptide (Figures S3a and 3(b)). Altogether, these results demonstrate that DRS-DA2 is structured in an *α*-helix and that DRS-DA2N seems better structured than DRS-DA2NEQ.

### 3.1. DRS-DA2 Induces Rapidly the Death of Immune Cells but Is Not Toxic for Erythrocytes or Epithelial Cells

**Figure 3:**
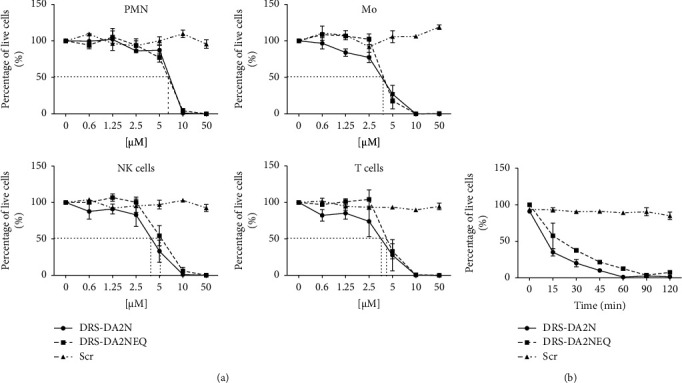
DRS-DA2N and DRS-DA2NEQ induce the death of human leukocytes. (a) Different populations of human leukocytes (PMN, Mo, NK, and T cells) were incubated for two hours at 37°C, 5% CO_2_ in the presence of various concentrations of DRS-DA2N, DRS-DA2NEQ, or Scr. The percentage of live cells was determined by flow cytometry. (b) Total human leukocytes were incubated in the presence of DRS-DA2N, DRS-DA2NEQ, and Scr at a concentration of 10 *µ*M for different time periods until 2 hours, and the percentage of live cells was determined by flow cytometry. Data represent three independent experiments run in triplicate.

The viability of different populations of human and mouse leukocytes (neutrophils, monocytes, NK, and T cells) was measured after incubating the cells for two hours at 37°C, 5% CO_2_ with various concentrations of DRS-DA2N and DRS-DA2NEQ, as well as Scr as control. Both peptides DRS-DA2N and DRS-DA2NEQ induced the death of human (Figure 3(a)) and mouse (Fig.  S4) neutrophils (PMN), NK, and T cells with an LC_50_ of around 5 *µ*M (Table 1), with DRS-DA2N being slightly more potent than DRS-DA2NEQ. As predicted, Scr did not induce the death of any of the human or mouse leukocyte populations.

To assess the kinetics of peptide-induced cell death, leukocytes were incubated with DRS-DA2N and DRS-DA2NEQ at a concentration of 10 *µ*M for different times, up until two hours. As observed in Figure 3(b), around 50% of the leukocytes were killed after 15 min of incubation with both DRS-DA2N or DRS-DA2NEQ. However, DRS-DA2N seems to induce the death of the cells in a slightly faster way.

**Figure 4:**
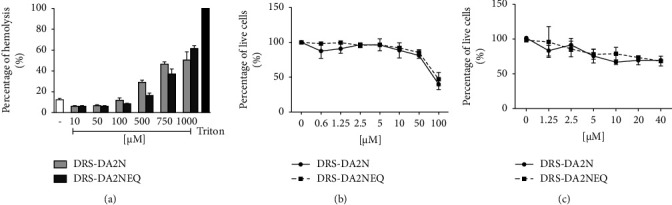
DRS-DA2N and DRS-DA2NEQ are not toxic for erythrocytes or epithelial cells. (a) Human erythrocytes in PBS were incubated with various concentrations of DRS-DA2N and DRS-DA2NEQ for one hour. Triton 1% was used as a positive control. Hemoglobin release was monitored by measuring the absorbance at 550 nm (mean of 3 independent experiments run in triplicate). (b) Human primary epithelial cells were incubated with DRS-DA2N and DRS-DA2NEQ at the indicated concentrations for 2 hours at 37°C, 5% CO_2_. The percentage of live cells was determined by flow cytometry. (c) A549 cells were incubated for 1 hour at 37°C, 5% CO_2_ in the presence of increasing concentrations of the peptides. The percentage of live cells was determined by the CCK8 assay. Experiments were run in duplicate.

To figure out if the induction of cell death by the peptides was restricted to leukocytes or affected other cell lineages, both peptides at different concentrations were incubated with human (Figure 4(a)) and mouse (Fig.  S5) red blood cells, human (Figure 4(b)) and mouse (Fig.  S5) primary epithelial cells, and the human tumoral epithelial cell line (A549) (Figure 4(c)). Interestingly, at the concentrations they induce the death of 100% of leukocytes (10 *µ*M), none of the peptides induced the death of any of these cell types.

These results suggest that DRS-DA2N and DRS-DA2NEQ preferentially induce the death of leukocyte lineage cells but not that of other cells, such as epithelial cells or erythrocytes.

### 3.2. DRS-DA2 Has a Broad-Spectrum Microbicidal Capacity

The antibacterial properties of DRS-DA2N, DRS-DA2NEQ, the control peptide Scr, and melittin as a reference pore-forming peptide were tested on a series of Gram-positive and Gram-negative strains. As presented in Table 2, both peptides exhibited a wide spectrum of action, which was however greater on Gram-negative than on Gram-positive bacteria even if some MICs ≤ 6.3 *µ*M were observed for *S. aureus* ATCC 6538 and ST 1065, *S. epidermidis* BM 3302, and *Enterococcus faecalis* CIP 103015. Interestingly, DRS-DA2N was generally four times more potent than DRS-DA2NEQ and also than melittin. Particularly, for the *E. coli β*-lactamase-resistant strain, the MIC observed for DRS-DA2N was 1.6 *µ*M, whereas the one for melittin was only 6.25 *µ*M. As predicted, Scr did not exhibit any bacterial activity.

As DRS-DA2N appeared to be more potent than DRS-DA2NEQ, its antibacterial activity was evaluated on various skin multiresistant strains and compared to reference antibiotics (Table 3). Interestingly, DRS-DA2N was active on multiresistant Gram-positive and Gram-negative strains and exhibited MICs lower than the reference antibiotics, even for Gram-positive strains. In the case of the clinical isolates of *P. acnes*, responsible for acne vulgaris, DRS-DA2N was less effective than the reference antibiotic doxycycline, yet it exhibited good activity.

**Figure 5:**
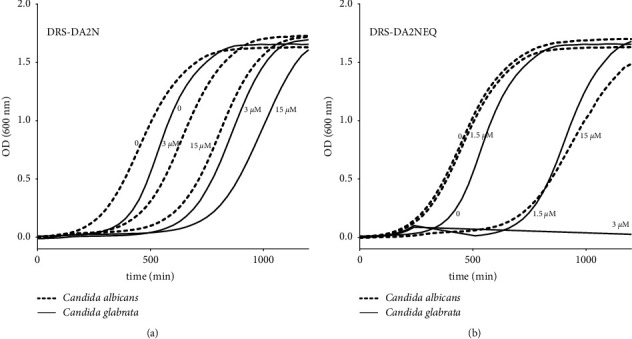
DRS-DA2N and DRS-DA2NEQ present antifungal activities. *C. albicans* and *C. glabrata* cells were grown for 48 h at 30°C and diluted into fresh YPD media containing DRS-DA2N (a) and DRS-DA2NEQ (b) at 1.5, 3, or 15 *µ*M. OD_600_ was determined every 15 min. The control corresponds to fungi without any peptide.

To further broaden the potential of DRS-DA2N and DRS-DA2NEQ to function as anti-microbial agents, we tested the antifungal activity of DRS-DA2N and DRS-DA2NEQ on *C. glabrata* CBS138 and *C. albicans* SC5314, two strains responsible for most bloodstream infections worldwide, associated with high mortality rates [23]. *C. glabrata* CBS138 and *C. albicans* SC5314 were grown in the presence of DRS-DA2N and DRS-DA2NEQ at various concentrations. Although the peptides did not inhibit the growth of *C. glabrata* CBS138 and *C. albicans* SC5314, the lag phase was extended for both fungal pathogens, as compared to the no-treatment culture. In the absence of either peptide, *C. albicans* started growing at 125 min, but in the presence of DRS-DA2N (3 or 15 *µ*M), the lag phase was extended to 250 min and that of *C. glabrata* up to 500 min (Figure 5(a)). In the presence of DRS-DA2NEQ, *C. albicans* also showed an extended lag phase and a reduced doubling time at 15 *µ*M. At that same concentration of DRS-DA2NEQ, *C. glabrata* showed an extended lag phase with no effect in doubling time (Figure 5(b)). These data indicate that both peptides DRS-DA2N and DRS-DA2NEQ have also antifungal activities.

**Figure 6:**
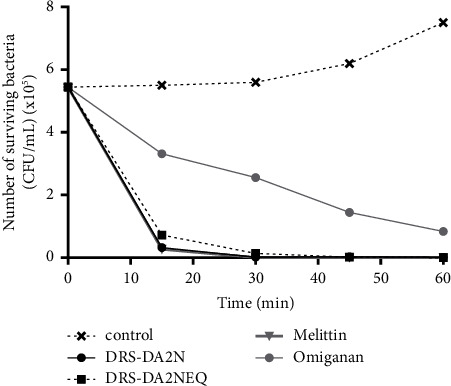
DRS-DA2N and DRS-DA2NEQ induce the death of Gram-negative bacteria rapidly. The death of bacteria was measured by the time-killing assay (TKA). Curves representing the amount of surviving bacteria (CFU/mL) in the absence or presence of DRS-DA2N, DRS-DA2NEQ, melittin, or omiganan at their MIC concentrations. The control corresponds to bacteria without any peptide.

We next investigated if, like leukocytes, bacteria were killed rapidly by DRS-DA2N and DRS-DA2NEQ. Time-kill curves of DRS-DA2N and DRS-DA2NEQ against *E. coli* ATCC8739 were performed. Curves representing the amount of surviving bacteria (CFU/mL) in the absence or presence of DRS-DA2N, DRS-DA2NEQ, melittin, or omiganan, an antibiotic used in the treatment of skin infections at their MIC concentrations, are presented in Figure 6. Interestingly, the growth of *E. coli* ATCC8739 was stopped rapidly by DRS-DA2N, DRS-DA2NEQ, and melittin, as all the bacteria were killed after only 15 min of incubation with either peptide at 37°C. In contrast, omiganan needed a much longer time to be active.

Altogether, these data show that DRS-DA2 peptides have rapid antibacterial and antifungal activities.

### 3.3. DRS-DA2 Has a Short Half-Life in the Presence of Serum

**Figure 7:**
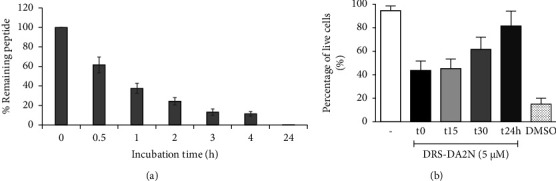
DRS-DA2N and DRS-DA2NEQ are resistant to enzymatic degradation for 15 minutes. (a) Quantification of the remaining peptide based on relative UV absorption at 220 nm of DRS-DA2N upon incubation with rat serum for different incubation time points (*n* = 3 independent experiments). (b) Recovered DRS-DA2N previously submitted to serum for different time points was incubated with mouse bone marrow cells for 2 hours, and cell viability was determined. Three independent experiments were run in triplicate.

Before assessing the anti-inflammatory activity of the peptides *in vivo*, the stability of DRS-DA2 to proteases was investigated. DRS-DA2N at a final concentration of 300 *µ*M was incubated in the presence of rat serum at 37°C for different times (0, 0.5, 1, 2, 3, 4, and 24 h) before being recovered and analyzed by RP-HPLC. Peptide degradation was followed at 220 and 280 nm. Quantification of the remaining peptide DRS-DA2N over time was performed by comparing the areas of the intact peptide DRS-DA2N with the sum of the areas of all the peptides (intact + degraded) present in the rat serum (Figure 7(a)). Each fraction was then collected and characterized by MALDI TOF/TOF (Fig.  S6). A 40% degradation of DRS-DA2N was observed after 30 min, and DRS-DA2N was totally degraded in the presence of serum for 24 h. MS/MS fragmentation of the degraded peptides, using MALDI-TOF/TOF, indicated a time-dependent N-terminal degradation (Fig.  S6).

Alternatively, DRS-DA2N (300 *µ*M) fractions previously incubated with serum for 0 (t0), 15 min (t15), 30 min (t30), or 24 h (t24 h) were recovered and incubated with cells from the mouse bone marrow for two hours at a concentration of 5 *µ*M. Cell death was evaluated by flow cytometry. As observed in Figure 7(b), no significant differences in the percentage of dead cells could be seen in the presence of DRS-DA2N t0 or t15. On the contrary, the percentage of dead cells started to decrease when cells were incubated with DRS-DA2N t30. When incubating cells with DRS-DA2N t24, the induction of cell death was almost completely abolished.

Both experiments indicated that DRS-DA2 degraded rapidly in the presence of proteases contained in the serum, as a consequence of which the peptide lost its immunosuppressive function.

### 3.4. DRS-DA2N Decreases Locally the Inflammation in a Mouse Model of Noninfectious Peritonitis

As DRS-DA2 induces the death of immune cells and bacteria rapidly, in less than 30 min, without affecting the viability of erythrocytes or epithelial cells, and since it also degrades rapidly in the presence of serum, we considered the possibility of using DRS-DA2 in inflammatory pathologies that could benefit of a local treatment, which would have the advantage to decrease the risks of adverse effects.

To validate the possibility of using the peptides *in vivo* as a local anti-inflammatory treatment, we used a mouse model of noninfectious peritonitis and injected the peptides directly in the peritoneal cavity. Basically, peritonitis was induced by injecting thioglycolate 3% in the peritoneal cavity. DRS-DA2N, DRS-DA2NEQ, Scr, or the vehicle (NaCl) were intraperitoneally (i.p.) injected 14, 19, and 24 h later. A group of mice injected with NaCl only was used as a control of the induction of peritonitis. Mice were sacrificed 12 h after the last injection of the peptides or vehicle. The effect of the peptides on the number of leukocytes in the peritoneal cavity, blood, and bone marrow was analyzed by flow cytometry, with monocytes, macrophages, and neutrophils being defined on the expression of CD11b, Ly6G, and F4/80 cell surface markers.

**Figure 8:**
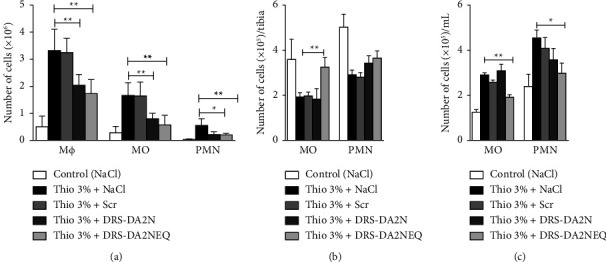
DRS-DA2N and DRS-DA2NEQ decrease the inflammation *in vivo* in a mouse model of noninfectious peritonitis. (a) Number of macrophages (CD11b^+^Ly6G^−^F4/80^+^), monocytes (CD11b^+^ Ly6G^−^ F4/80^−^), and neutrophils (CD11b^+^Ly6G^+^) recruited to the peritoneal cavity. (b) Number of monocytes and neutrophils in the bone marrow (BM) (c) and blood. Data are given as the mean ± SEM of 3 independent experiments run with 6 mice in each group. MΦ, macrophages; Mo, monocytes; PMN, neutrophils. ^*∗*^*P* ≤ 0.01; ^*∗∗*^*P* ≤ 0.01.

The injection of thioglycolate 3% and the peptide vehicle (NaCl) increased the level of monocytes, macrophages, and neutrophils in the peritoneal cavity, compared to the control group (NaCl only). Three i.p. injections of DRS-DA2 after the thioglycolate 3% injection resulted in a significant decrease in the number of monocytes, macrophages, and neutrophils in the peritoneal cavity of the mice compared to the group of mice injected with thioglycolate 3% and vehicle (NaCl) or Scr (Figure 8(a)).

To investigate whether the lesser number of leukocytes observed in the peritoneal cavity after treatment with DRS-DA2N and DRS-DA2NEQ was restricted to a local environment (peritoneal cavity) or was systemic, we evaluated the number of inflammatory cells in blood and in the bone marrow. As expected, induction of peritonitis led to an egress of monocytes and neutrophils from the bone marrow (Figure 8(b)) and an increase in blood (Figure 8(c)). Interestingly, this egress of inflammatory cells from the bone marrow and this increase in blood observed after induction of peritonitis and treatment with DRS-DA2N or Scr were conserved, whereas the egress from the bone marrow and the increase of monocytes in the blood were reduced after induction of peritonitis and treatment with DRS-DA2NEQ (Figures 8(b) and 8(c)). Thus, these results indicate that the i.p. treatment with both peptides reversed the increase of inflammatory cells in the peritoneal cavity after induction of peritonitis. However, whereas the effect of DRS-DA2N was restricted to the peritoneal cavity, DRS-DA2NEQ seemed to have a more systemic effect, as there is a significant diminution of the egress of monocytes in the bone marrow and of the increase of monocytes in blood.

### 3.5. DRS-DA2 Affects the Integrity of the Membrane of Immune Cells and Bacteria

The rapid killing action of the peptides (under 30 min) of both leukocytes and bacteria supports evidence that the peptides induced the death of cells and bacteria in a like manner, i.e., by a nonreceptor-mediated mechanism.

**Figure 9:**
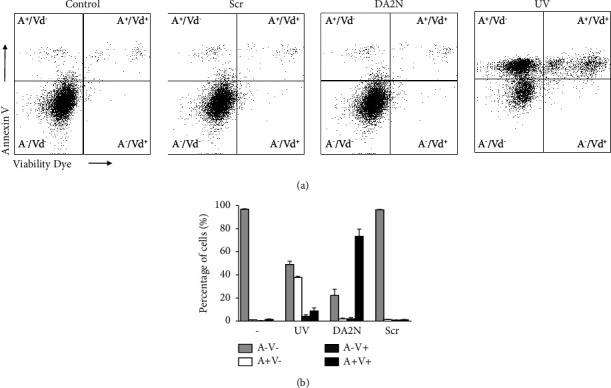
DRS-DA2N induces the death of immune cells by membrane disruption. Jurkat E6-1 cells were incubated for two hours with DRS-DA2N, Scr, or PBS or exposed to UV for 10 min and put to rest for 4 hours, 5% CO_2_ at 37°C, before being stained with annexin V and the viability dye eFluor 780. (a) Representative flow cytometry analysis of Jurkat E6-1 cells double stained with annexin V (A) and the viability dye eFluor 780 (V). (b) Percentage of cells A^−^V^−^, A^+^V^−^, A^−^ V^+^, and A^+^V^+^ after the different treatments. Two experiments were run in triplicate.

As a first step, we investigated if the peptides induced the death of immune cells by directly disrupting the cytoplasmic membrane (necrosis) or by the more regulated process of apoptosis, where membrane integrity is maintained. First, to assess that DRS-DA2 induced the death of Jurkat E 6-1 cells similarly to primary leukocytes, Jurkat E 6-1 cells were incubated with DRS-DA2N or Scr at various concentrations, and viability was measured. Similarly, to what was observed on primary leukocytes, DRS-DA2N induced the death of Jurkat cells after 2 hours with an LC_50_ of 4 *µ*M (Figure S7). Then, to differentiate the two death pathways, Jurkat E 6-1 cells were incubated for 2 hours with DRS-DA2N or Scr at a concentration of 5 *µ*M or with the vehicle (PBS) as a negative control, and the cells were stained with a viability dye (eFluor 780, V) and annexin V (A) (Figure 9(a)). A positive control of apoptosis was generated by exposing the cells to UV (1 mJ/cm^2^) for 10 min and incubating them for 4 hours at 37°C, 5% CO_2_ prior to staining for annexin binding and viability. After incubation with DRS-DA2N, around 70% of the remaining cells were annexin and eFluor 780 positive (A^+^V^+^), indicating they died of necrosis (Figure 9(a)). As expected, around 40% of the UV-irradiated cells were in the apoptotic cells' quadrant (A^+^V^−^). Like untreated cells (negative control), hardly any cell death (<4%) was observed after the treatment with Scr, as more than 96% of cells were alive (A^−^V^−^) (Figure 9(b)). These results suggest that DRS-DA2N could induce the death of immune cells by permeabilizing the membrane leading to the loss of membrane integrity.

**Figure 10:**
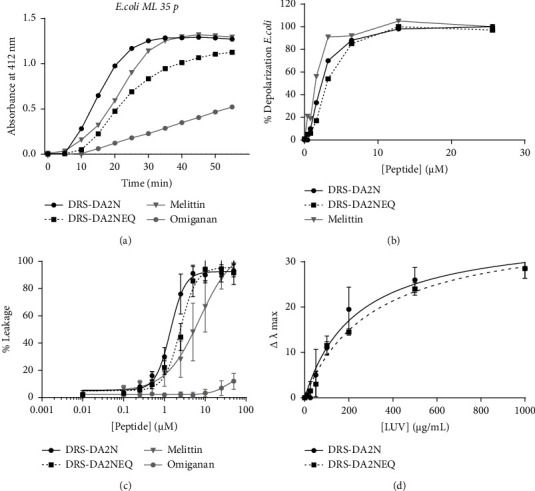
Bacterial membrane permeabilization. (a) *E. coli* ML35p inner membrane permeabilization by DRS-DA2 peptides at a 2 × MIC concentration in comparison with melittin or omiganan revealed by hydrolysis of ONPG. (b) Depolarization effects of DRS-DA2N, DRS-DA2NEQ, and melittin on the *E. coli* cytoplasmic membrane. (c) ANTS/DPX assay with DRS-DA2 peptides in comparison with melittin and omiganan. (d) Tryptophan blue shift (Δ*λ*max) in the presence of liposomes produced from *E. coli* ML35p and DRS-DA2N or DRS-DA2NEQ peptides.

We next investigated if DRS-DA2N and DRS-DA2NEQ also permeabilized the membrane of the bacteria. The extent of membrane permeabilization due to the peptides' activity was evaluated by using the ONPG test on the *E. coli* ML35p strain. This mutant is constitutive for cytoplasmic *β*-galactosidase, lacks lac permease, and expresses a plasmid-encoded periplasmic *β*-lactamase. Since there is no lactose permease in this mutant, ONPG cannot cross its inner membrane to be cleaved by cytoplasmic *β*-galactosidase to ONP unless permeabilization of the inner membrane occurs. ONPG cleavage produces a color change that can be measured spectrophotometrically at 420 nm [24]. DRS-DA2N and DRS-DA2NEQ, as well as melittin, induced inner membrane permeabilization, whereas, even after 60 min, omiganan did not reach the plateau (Figure 10(a)), indicating that omiganan seems to adopt another mode of action. The slight differences observed between the DRS-DA2 peptides and melittin were not significant because they probably were due to minor differences in concentrations, taking account of the very small concentrations used in this test.

The cytoplasmic membrane depolarization activity of the peptides was determined by using the membrane potential-sensitive fluorescent dye diSC_3_-5 on *E. coli* membranes [25]. If bacteria permeabilization and disruption of the cytoplasmic membrane occur following contact with the peptides, an enhancement in fluorescence will be observed as a consequence of the dispersion of the fluorescent probe in the medium. Our first results on *E. coli* showed that DRS-DA2N and DRS-DA2NEQ, as well as melittin, induced rapid depolarization within 10 min (Figure 10(b)).

To go further in the study of DRS-DA2 peptide interaction with lipids, we used liposomes as a reliable model to characterize AMP action on a membrane [26]. The permeabilization of LUV membranes induced by the peptides was assessed by using the ANTS/DPX assay that can be used to measure the leakage of small molecule probes from lipid vesicles [27]. ANTS (8-aminonaphthalene-1,3,6-trisulfonic acid, disodium salt) is a highly negatively charged dye used together with the fluorescent quencher DPX (*N,N′*-p-xylene-bis (pyridinium bromide)) for the studies of membrane fusion or permeability. The mixture of ANTS-DPX is minimally fluorescent initially but becomes increasingly fluorescent upon dilution by leakage into the buffer. To mimic *E. coli* membranes, a mixture of POPE and POPG (75 : 25) LUVs was used. In this model system, DRS-DA2N and DRS-DA2NEQ were shown to act similarly as melittin, allowing a rapid increase in fluorescence when enhancing the peptide's concentration (Figure 10(c)). The LIC_50_ (the peptide concentration required to induce 50% leakage) of DRS-DA2N and DRS-DA2NEQ was similar, whereas melittin was slightly less active (Table 4). As previously reported by Melo et al. [28], omiganan did not induce any leakage.

The tryptophan fluorescence spectrum depends on the tryptophan environment, giving information on aqueous or lipid surroundings of the indole nucleus. As the DRS-DA2 peptides contain tryptophan in position 3, the study of the tryptophan fluorescence emission spectra could give indications of the peptides' ability to insert in membranes that would be characterized by a blue shift of *λ*_max_. The tryptophan fluorescence spectra of DRS-DA2N and DRS-DA2NEQ in aqueous buffer had a maximum emission *circa* 355 nm. Addition of increasing concentration of LUVs made with lipids extracted directly from *E. coli* ML35p showed a large blue shift (30-35 nm) in the emission maxima of the DRS-DA2N and DRS-DA2NEQ together with an enhancement of emission intensity maxima, indicating the incorporation of the tryptophan aromatic ring into the hydrophobic medium of lipids (Figure 10(d)).

Altogether, these results indicate that similarly to what is observed for immune cells, the induction of bacterial death by DRS-DA2 involves a strong disturbance of the membrane.

## 4. Discussion

In this study, a new member of the demarseptin family, DRS-DA2, exhibiting in vitro antimicrobial and immunosuppressive activities at a micromolar range and in vivo anti-inflammatory activity; both within 30 min; was isolated from the endemic Mexican tree frog *Pachymedusa dacnicolor*. Among the recorded 3569 AMPs (APD3, https://aps.unmc.edu/) [29], there are only 32 peptides reported with both antibacterial and anti-inflammatory activities. Most of these peptides exert their anti-inflammatory activity by inhibiting the production of inflammatory cytokines and chemokines, such as TNF-alpha, IL-6, IL-1-beta, or CCL2, or increasing the production of anti-inflammatory cytokines such as IL-10 [30], by inhibiting interaction of LPS to toll-like receptors [31] or the production of nitric oxide [32]. To our knowledge, DRS-DA2 is the first AMP to induce specifically the death of immune cells and thus regulating locally the pool of leukocytes at the site of inflammation in an *in vivo* inflammatory model.

Interestingly, three different forms of this peptide, DRS-DA2NEQ, DRS-DA2NE, and DRS-DA2N, appear to coexist naturally. The existence of such truncated peptides has already been reported in the skin secretions of *Phasmahyla jandaia* where bradykinin appears as a full (RPPGFSPFR) or a C-terminal truncated form (RPPGFSPF) [33], though this does not completely refute a possible degradation of the sample, leading to the loss of residues in the C-terminal part of the peptide DRS-DA2. Although this is unlikely, since all the peptides present in the other fractions are intact. The full-length peptide was predicted by Wechselberger in 1998 but had never been isolated until now [7]. DRS-DA2NEQ and DRS-DA2NE present a net charge of +4, while DRS-DA2N has a net charge of +5 and is thus slightly more charged than most of the recorded AMPs having a net charge between +2 and +4. Like numerous AMPs, and particularly those of the dermaseptin family, DRS-DA2 is structured in the alpha-helix in contact with micelles that mimic amphipathic properties of the membranes lipid bilayer, with the C-terminal region of the peptide rather unstructured, and exhibits a pronounced amphipathic character (Figures 2(a) and 2(b)). Interestingly, the short form of the peptide DRS-DA2N presents a higher percentage of helicity, indicating that it seems to be better structured. These differences in terms of the net charge and structural conformation seem to play a role in the activity of the peptide as DRS-DA2N exhibits slightly better antimicrobial and immunosuppressive activities than DRS-DA2NEQ (Tables 1 and 2). These observations are consistent with the literature as the antibacterial mechanism of action of these kinds of peptides depends on the positive net charge and alpha-helix conformation of the peptide [34]. The detailed mechanism by which cationic AMPs inhibit bacterial growth leading to their death has not yet been totally described. However, it is now known that most of these peptides interact through their N-terminal region with the membrane before disrupting and permeabilizing it, whereby several biophysical properties of the peptide (amphipathicity, net charge, hydrophobicity, structural conformation, or flexibility) and also of the membrane (charge, conformation, curvature, flexibility, or composition) are implicated [34, 35]. In the present study, the possible mechanism of bacterial death induced by DRS-DA2 was evaluated by various biophysical techniques and showed that the peptide disrupted the bacterial membrane within 30 minutes. As the immunosuppressive effect was also observed within 30 minutes after incubating leukocytes with DRS-DA2, we hypothesized that the peptide would induce the death of the cells by necrosis, which is a mechanism of death very similar to the bacterial membrane disruption and which occurs rapidly. Indeed, using necrotic and apoptotic markers, we showed that the peptide induced necrosis rather than apoptosis.

Cancer cell membranes differ from primary cells by exhibiting more negatively charged components, greater fluidity, and cell surface area [36]. These properties play an important role in the induction of cancer cell death by enabling cationic peptides to interact better with the membrane of cancer cells than with that of primary cells. However, we could not note significant differences in the induction of death of primary *versus* cancer cells (Figures 3(a), S7, 4(b) and 4(c)), indicating that the biophysical properties mentioned above are not sufficient for DRS-DA2 to induce cell death. Moreover, as far as we are aware, this is the first report documenting a peptide that induces specifically the death of one type of cells that are not tumor cells, without affecting other types of eukaryotic cells at this range of concentration. The exact molecular mechanisms by which DRS-DA2 induces bacteria and leukocyte death, as well as the difference in sensitivity of DRS-DA2 on leukocytes and other eukaryotic cells, are for the moment, not fully understood. To clarify these points, further investigations will be needed.

DRS-DA2 also displays a different sensitivity on Gram-negative and Gram-positive bacteria by exhibiting an enhanced effect on Gram-negative bacteria, with a MIC and an MBC significantly lower. These findings are quite unusual, as observed by Malanovic [34]. Indeed, it has been demonstrated that the peptidoglycan composition of the outer membrane wall of Gram-positive bacteria would function as a cell wall sponge, attracting peptides to the surface of the bacterial lipid membrane, thus potentiating the action of antimicrobial peptides by increasing their local concentration at the level of the bacterial membrane.

Nevertheless, we showed that DRS-DA2N inhibited the growth of the skin multiresistant Gram-positive strain *S. aureus* ATCC 700699 (MecA +), frequently responsible for nosocomial infections, and is though better than the reference antibiotic (10 times better). DRS-DA2N also inhibited the growth of the other skin Gram-positive bacteria *Propionibacterium acnes*, responsible for acne vulgaris, which is one of the most common skin diseases affecting more than 630 million people in the word [37]. However, even if DRS-DA2 exhibited an MIC higher than the reference antibiotic doxycycline, it still presented a good activity. It should be noted that doxycycline is used as oral treatment and can result in severe aplastic anemia [38], whereas DRS-DA2 could be used topically and locally. DRS-DA2N also inhibited the growth of two Gram-negative skin bacteria responsible for nosocomial infections, *S. epidermidis* ST20140436 (MecA +) and *E. coli* OXA-48 (carbapenemase) with MICs lower than the reference antibiotic. DRS-DA2 was also efficient against two candida species, *Candida glabrata* CBS138, which present an innate resistance to azole antimycotic therapy that is very effective in treating infections caused by other Candida species, and *Candida albicans.* Most Candida infections are benign, though they can lead to death in immunocompromised hosts. Until recently, *C. glabrata* was considered a relatively nonpathogenic fungal organism. However, with the increased use of immunosuppressive agents, mucosal and systemic infections caused by *C. glabrata* have raised significantly [39].

Most treatments used in inflammatory diseases are small drugs that interact with various pharmacological targets that exhibit poor selectivity and high toxicity that are associated with numerous side effects. Most of them present systemic effects, which in long-term therapies usually weaken the ability of the immune system to combat infections [40]. Thus, the use of peptides, and in particular, peptides exhibiting dual action, i.e., anti-inflammatory and antibacterial, could be an alternative treatment to small molecules. Compared to small molecules, peptides present high potency and selectivity, low toxicity, low risk of drug interaction, and low accumulation in tissue. Nevertheless, poor metabolic stability and rapid clearance of bioactive peptides are usually seen as a restriction in their use [40, 41]. As expected for a peptide, DRS-DA2 demonstrated poor metabolic stability as it started to degrade within one hour. However, we also showed that it induced the death of bacteria and immune cells in less than 30 min. Thus, this rapid clearance should not affect its antibacterial and immunosuppressive activities *in vivo* after a local application. Furthermore, it would have the advantage of minimizing the dissemination of the peptide in the body, thus decreasing the risks of side effects. Moreover, the fact that DRS-DA2 exhibits a rapid action is very promising as less peptide would be necessary to kill bacteria in comparison with slow-acting peptides or small molecules (time-kill greater than 2 h) since bacteria are able to double in the number every 20 min [42]. We showed that the local application (i.p. injection) of DRS-DA2 after induction of noninfectious peritonitis reversed the number of leukocytes in the peritoneal cavity. Additional *in vivo* experiments would be necessary to confirm if the decrease observed is due to the death of leukocytes as hypothesized or to another effect on leukocytes, such as inhibition of migration. Notably, the short form DRS-DA2N exhibited a local effect, as no modulation of the number of leukocytes was observed in the bone marrow or in blood after peritonitis induction. Furthermore, as the *in vivo* effect of the peptide was validated in a short-term inflammatory model, it would be of great interest to investigate its effect on a more relevant inflammatory model, such as a chronic skin inflammatory disease, to ensure that the death induction of leukocytes does not trigger proinflammatory signals, such as the production of inflammatory cytokines, angiogenesis, or vasodilation.

## 5. Conclusions

In conclusion, to our knowledge, DRS-DA2 is the first AMP to exhibit antimicrobial activity combined with the capacity to regulate the pool of inflammatory cells by inducing specifically the death of leukocytes at a concentration within the micromolar range without being toxic to other eukaryotic cells such as erythrocytes or primary and transformed epithelial cells at that concentration. As the short form DRS-DA2N is slightly more potent at inducing the death of bacteria and leukocytes, as well as presenting a capacity to regulate the pool of inflammatory cells locally in the inflammatory model of peritonitis, it could be a promising therapeutic agent in the treatment of inflammatory diseases, and particularly, in skin inflammatory diseases.

## Figures and Tables

**Table 1:** LC_50_ (*µ*M) of DRS-DA2N and DRS-DA2NEQ.

	LC_50_ (*µ*M)			
	DRS-DA2N		DRS-DA2NEQ	
	Human cells	Mouse cells	Human cells	Mouse cells
PMN	7.5	6	7	7.5
Mo	4	5	4	7
T cells	4	3	5	5
NK cells	4	3	5	4

**Table 2:** Antimicrobial activities of DRS-DA2N and DRS-DA2NEQ on Gram-negative and Gram-positive strains.

	Antimicrobial activity: MIC/MBC (pM)							
	DRS-DA2N		DRS-DA2NEQ		SAT		Melittin	
	MIC	MBC	MIC	MBC	MIC	MBC	MIC	MBC
*Gram-negative strains*								
*E. coli* ATCC 8739	0.8	0.8	1.6	1.6	>50	>50	3.2	3.2
*E. coli* K12	3.2	6.3	12.5	12.5	n.d	n.d	12.5	12.5
*E. coli* ML35p	0.8	0.8	1.6	1.6	n.d	n.d	12.5	12.5
*E. coli* P7 (BLSE)^*∗*^	1.6	12.5	12.5	50	n.d	n.d	6.3	6.3
*P. aeruginosa* ATCC 9027	3.2	3.2	6.3	6.3	>50	>50	6.3	6.3
*P. aeruginosa* ATCC 27853	12.5	50	50	>100	n.d	n.d	50	>50
*K. pneumoniae* CIP 52.211	0.8	0.8	0.8	0.8	n.d	n d	12.5	12.5
*K. oxytoca* CIP 7932	3.2	6.3	12.5	>100	n.d	n.d	zs	No
*S. enterica* CIP 8297	12.5	25	50	100	n.d	n.d	50	>50
*Y. ruckeri* ATGG 29473	3.2	12.5	25	>100	n.d	n.d	6.3	12.5

*Gram-positive strains*								
*S. aureus* ATCC 6538	1.6	3.2	6.3	12.5	>50	>50	6.3	6.3
*S. aureus* MRSA^*∗*^	25	>50	100	>100	n.d	n.d	6.3	12.5
*S. aureus* ST 1065	6.3	6.3	12.5	25	n.d	n.d	10	10
*S. epidermidis* BM 3302	6.3	25	12.5	>50	n.d	n.d	10	10
*L. monocytogenes* SOR 100	25	25	100	>100	n.d	n.d	1.5	3.2
*E. faecalis* CIP A186	50	>50	100	>100	n.d	n.d	12.5	12.5
*L. garvieae* ATCC 43921	25	50	100	>100	n.d	n.d	6.3	25
*E. faecalis* CIP 103015	6.3	6.3	12.5	12.5	>50	>50	1.6	1.6
*K. rhizophila* ATCC 9341	0.8	1.6	1.6	1.s	n.d	n.d	0.8	0.8

^*∗*^Multiresistant strains.

**Table 3:** Antibacterial activities of DRS-DA2N on some skin multiresistant bacteria in comparison with reference antibiotics.

Bacterial strains	DRS-DA2N (*µ*M)	ATB (*µ*M)
*S. aureus* ATCC 700699 (MecA+)	>5.2	>43.8^a^
*S. epidermidis* ST20140436 (MecA+)	5.2	>43.8^a^
*E. coli* OXA-48 (carbapenemase)	5.2	>43.8
*Acinetobacter johnsonii* (carbapenemase)	1.3	>29.3^b^
*Propionibacterium acnes* (clinical isolate)	1.3	0.13^c^
*Propionibacterium acnes* (clinical isolate)	0.6	0.27^c^

ATB: reference antibiotics, a: amoxicillin, b: ceftazidime, and c: doxycycline.

**Table 4:** Membrane perturbation studied by the ANTS/DPX assay from POPE/POPG LUVs.

Peptides	LIC_50_ (*µ*M)
DRS-DA2N	1.5 ± 0.3
DRS-DA2NEQ	2.3 ± 0.5
Melittin	7.2 ± 3.5
Omiganan	No leakage

LIC_50_ obtained from Figure 10(c).

## Data Availability

The data used to support the findings of this study are included within the article or within the supplementary information file.

## Abbreviations

AMP: Antimicrobial peptides
BM: Broth medium
CD: Circular dichroism
DMPC: 1,2-Dimyristoyl-sn-glycero-3-phosphatidylcholine
DMPG: 1,2-Dimyristoyl-sn-glycero-3-phosphatidylglycerol
RP-HPLC: Reversed-phase high pressure liquid chromatography
MALDI-TOF: Matrix-assisted laser desorption/ionization-time-of-flight
MERS-CoV: Middle East respiratory syndrome-related coronavirus
MIC: Minimal inhibitory concentration
NK: Natural killer
NMR: Nuclear magnetic resonance
ONPG: 2-Nitrophenyl-*β*-D-galactopyranoside
PBMC: Peripheral blood mononuclear cells
PMN: Polymorphonuclear
SARS-CoV: Severe acute respiratory syndrome coronavirus
UV: Ultraviolet.

## Supplementary Materials

Supplementary Materials Supplementary 1: Figure S1: isolation and purification of a new immunosuppressor peptide, dermaseptin DRS-DA2. Supplementary 2: Table S1: NMR and refinement statistics for the structure calculation of DRS-DA2N bound to SDS micelles (12 conformers). Supplementary 3: Figure S2: far-UV CD spectra of DRS-DA2N, DRS-DA2NEQ, melittin, and Scr in Tris 10 mM. Supplementary 4: Figure S3: NMR conformational study of DRS-DA2N bound to SDS micelles. Supplementary 5: Figure S4: DRS-DA2N and DRS-DA2NEQ induce the death of mouse leukocytes. Supplementary 6: Figure S5: DRS-DA2N and DRS-DA2NEQ are not toxic for erythrocytes or epithelial cells. Supplementary 7: Figure S6: stability of the peptide DRS-DA2N in rat serum. Supplementary 8: Figure S7: DRS-DA2N induces the death of Jurkat cells.
