# Supplementary material for: Characterization of a New Immunosuppressive and Antimicrobial Peptide, DRS-DA2, Isolated from the Mexican Frog, Pachymedusa dacnicolor
Source: Int J Inflam. 2024 Jan 13;2024:2205864. doi: 10.1155/2024/2205864 (PMC10799709; doi:10.1155/2024/2205864)
Supplement: Supplementary Materials — Supplementary 1: Figure S1: isolation and purification of a new immunosuppressor peptide, dermaseptin DRS-DA2. Supplementary 2: Table S1: NMR and refinement statistics for the structure calculation of DRS-DA2N bound to SDS micelles (12 conformers). Supplementary 3: Figure S2: far-UV CD spectra of DRS-DA2N, DRS-DA2NEQ, melittin, and Scr in Tris 10 mM. Supplementary 4: Figure S3: NMR conformational study of DRS-DA2N bound to SDS micelles. Supplementary 5: Figure S4: DRS-DA2N and DRS-DA2NEQ induce the death of mouse leukocytes. Supplementary 6: Figure S5: DRS-DA2N and DRS-DA2NEQ are not toxic for erythrocytes or epithelial cells. Supplementary 7: Figure S6: stability of the peptide DRS-DA2N in rat serum. Supplementary 8: Figure S7: DRS-DA2N induces the death of Jurkat cells. [file 2205864.f1.docx]

**Supplementary Materials**

**Absorbance (280nm)**

**Figure S1: Isolation and purification of a new immunosuppressor peptide, the dermaseptin DRS-DA2.** The Crude extract was fractionated on a Sephadex G-50 column, and the fractions obtained were tested for a death cell induction effect (insert) by incubating the fractions in the presence of leucocytes at various concentrations for two hours at 37°C, 5% CO_2_.

**Figure S2: Far-UV CD spectra of DRS-DA2N, DRS-DA2NEQ, Melittin, and Scr in Tris 10mM.**

**Experimental restraints**

Number of nOe-derived distance restraints 324

Ambiguous 6

Unambiguous 318

Intra-residue 121

Sequential (|*i – j*| = 1) 102

Medium-range (2 ≤ |*i – j*| ≤ 5) 95

Long-range (|*i – j*| > 5) 0

Number of dihedral angle restraints 50

φ 25

ψ 25

**Structural statistics**

Violations

No. distance restraints

> 0.5 Å 0 ± 0

> 0.3 Å 0 ± 0

> 0.1 Å 2.7 ± 0.9

No. dihedral angle restraints (> 5°) 0 ± 0

RMS deviations from ideal geometry

Bond (Å) 0.003

Angle (°) 0.43

Improper (°) 1.31

RMS deviations on restraints

Distance (Å) 0.02

Dihedral (°) 0.11

Structural dispersion (residues 2-26)

Backbone (Å) 0.5

Heavy atoms (Å) 1.1

Ramachandran plot (residues 2-26)

Most favored 100%

Additionally allowed 0%

Generously allowed 0 %

Disallowed regions 0 %

MolProbity all-atom Clashscore 14.89

**Table S1.** NMR and refinement statistics for the structure calculation of DRS-DA2N bound to SDS micelles (12 conformers).

**Figure S3. NMR conformational study of DRS-DA2N bound to SDS micelles.** **a/**Chemical shift deviations (CSD) of Calpha resonances with respect to random coil values. Large positive deviations (CSD > 1.0 ppm) indicate helical conformations in the region L2-A26. **b/** Backbone superimposition of the calculated NMR ensemble (12 lowest-energy conformers) over residues L2-A26.

**NK cells**

**PMN**

**Mo**

**T cells**

**Figure S4: DRS-DA2N and DRS-DA2NEQ induce the death of mouse leukocytes.** Different populations of mouse leukocytes (PMN, Mo, NK, and T cells) were incubated for two hours at 37°C, 5% CO_2_ in the presence of various concentrations of DRS-DA2N or DRS-DA2NEQ. The percentage of live cells was determined by flow cytometry. Data represent three independent experiments run in triplicate.

**a**

**b**

**Figure S5: DRS-DA2N and DRS-DA2NEQ are not toxic for erythrocytes or epithelial cells. a/** Mouse erythrocytes in PBS were incubated with various concentrations of DSR-DA2N and DRS-DA2NEQ for one hour. Triton 1% was used as positive control. The hemoglobin release was monitored by measuring the absorbance at 550 nm (mean of 3 independent experiments run in triplicate). **b/** Mouse primary epithelial cells were incubated with DRS-DA2N and DRS-DA2NEQ at various concentrations for 2 hours at 37°C, 5% CO_2_. The percentage of live cells was determined by flow cytometry.

**Figure S6: Stability of the peptide DRS-DA2N in rat serum.** RP-HPLC chromatogram of the injected DRS-DA2N after 0, 0.5, 1, 2, 3, 4, and 24 h of incubation in rat serum. Arrows show the sense of variation of the absorbance at 220 nm. Fractions were collected, and peptides were characterized by MALDI-TOF/TOF. m/z ratios of the monoprotonated peptides are indicated, as well as the corresponding determined sequences.

**Figure S7: DRS-DA2N induces the death of Jurkat cells.** Jurkat cells were incubated for two hours at 37°C, 5% CO_2_ in the presence of various concentrations of DRS-DA2N or Scr. The percentage of live cells was determined by flow cytometry.
